# Socio-economic, behavioural, (neuro)psychological and clinical determinants of HRQoL in people living with HIV in Belgium: a pilot study

**DOI:** 10.7448/IAS.16.1.18643

**Published:** 2013-12-12

**Authors:** Sophie Degroote, Dirk P. Vogelaers, Peter Vermeir, An Mariman, Ann De Rick, Bea Van Der Gucht, Jolanda Pelgrom, Filip Van Wanzeele, Chris Verhofstede, Dominique M. Vandijck

**Affiliations:** 1 Department of General Internal Medicine, Infectious Diseases and Psychosomatics, Ghent University Hospital, Ghent, Belgium; 2 Faculty of Medicine and Health Sciences, Department of Internal Medicine, Ghent University, Ghent, Belgium; 3 AIDS Reference Laboratory Ghent, Faculty of Medicine and Health Sciences, Department of Clinical Chemistry, Microbiology and Immunology, Ghent University, Ghent, Belgium; 4 Faculty of Medicine and Health Sciences, Department of Public Health, Ghent University, Ghent, Belgium; 5 Department of Health Economics & Patient Safety, Hasselt University, Faculty of Business Economics, Diepenbeek, Belgium

**Keywords:** HIV, AIDS, quality of life, MOS-HIV, psychosocial, outcome

## Abstract

**Introduction:**

Due to highly active antiretroviral therapy (HAART), HIV-1 infection has evolved from a lethal to a chronic disease. As such, health-related quality of life (HRQoL) has become an important outcome variable. The purpose of this study was to identify socio-economic, behavioural, (neuro)psychological and clinical determinants of HRQoL among people living with HIV (PLHIV).

**Methods:**

This study was conducted between 1 January and 31 December 2012 at the AIDS Reference Centre of Ghent University Hospital, a tertiary care referral centre in Belgium. Validated self-report questionnaires were administered to collect socio-demographic data, to assess HRQoL (Medical Outcomes Study-HIV), depressive symptoms (Beck Depression Inventory-II) and adherence to HAART (Short Medication Adherence Questionnaire) and to screen for neurocognitive dysfunction.

**Results:**

A total of 237 people participated, among whom 187 (78.9%) were male. Mean age was 45.8±10.7 years and 144 (63.7%, 144/226) participants were homosexual. Median physical and mental health score (PHS, MHS) were 55.6 (IQR 48.2–60.6) and 52.0 (IQR 44.2–57.9), respectively. Multivariable regression analysis revealed that incapacity to work, depressive symptoms, neurocognitive complaints (NCCs), dissatisfaction with the patient–physician relationship and non-adherence were all negatively associated with HRQoL.

**Conclusions:**

Socio-economic (work status), behavioural (adherence) and (neuro)psychological (depressive symptoms, NCCs) determinants independently impact HRQoL among this cohort of PLHIV. Clinical parameters (viral load, CD4 cell count) were not independently associated with HRQoL.

## Introduction

In Belgium, 12,600 people living with HIV (PLHIV) are medically followed, and there is a stable incidence rate of three new diagnoses per day [1]. Highly active antiretroviral therapy (HAART) therapy is reimbursed by health insurance, and more than 80% of the people on HAART have an undetectable viral load [2]. Worldwide, similar significant improvements in virological control through HAART emerge, and HIV has developed into a chronic disease with reasonable life expectancy [3]. Hence, the prevalence of PLHIV still increases [4]. As a result, concerns about lifelong medication use as well as physical and mental consequences of a chronic illness became important outcome indicators. In HIV treatment decisions, the impact on health-related quality of life (HRQoL) is a particular point of interest [5]. Maximizing HRQoL is a key goal in HIV care and, therefore, it is necessary to identify which factors influence HRQoL. Changing or managing these factors by means of targeted interventions should be the focus of healthcare managers and policy makers.

Studies investigating determinants of HRQoL found associations with clinical [6–9], socio-economic [10–12], psychological [13–16] and behavioural [17–20] parameters. Those studies mostly examined a specific and limited set of parameters within their field of interest. However, few studies [21–23] evaluated parameters from different categories simultaneously and none of them included neuropsychological parameters. As such, the possible distinctive influence of neurocognitive problems on HRQoL remains unclear.

This study is the first to investigate HRQoL in Belgium, and the first to incorporate neuropsychological parameters in a broad and detailed set of possible determinants of HRQoL.

## Methods

### Setting

The study was conducted at the AIDS Reference Centre of Ghent University Hospital (GUH), one of nine tertiary care referral centres in Belgium involved in the medical treatment and emotional and social support for PLHIV. One thousand three-hundred PLHIV are currently followed by a multidisciplinary team of physicians, (social) nurses, psychologists and psychiatrists. The collaborating AIDS Reference Laboratory is responsible for HIV diagnosis (serology, viral load, resistance, genotyping) and data collection of blood values.

### Participants

To obtain a representative sample of the HIV population seen in the ARC, all PLHIV consulting the ARC during the study period were eligible to participate. There were no constraints concerning age, time since diagnosis, treatment or other variables. Treating physicians recruited participants during a routine patient visit via informed consent, available in Dutch, French and English. The study was approved by the Ethics Committee of GUH and recruitment of participants started on 1 January 2012.

### Instruments

Patients were asked to fill in a self-developed and validated self-report questionnaire and to participate in a short interview with a nurse. Socio-demographic and socio-economic questions, as well as three validated instruments (Medical Outcomes Study-HIV – MOS-HIV, Short Medication Adherence Questionnaire – SMAQ and Beck Depression Inventory-II – BDI-II) were incorporated in the bundle.

To evaluate HRQoL, the MOS-HIV [24] was used. This instrument contains 35 items, which lead to 11 subscale scores between 0 and 100 (health perceptions, pain, physical functioning, social functioning, role functioning, mental functioning, energy, cognitive functioning, health distress, quality of life and health transition). Two final scores are then calculated: the physical health score (PHS) and the mental health score (MHS).

In addition, participants were asked to fill in the six-item SMAQ [25] and the 21-item BDI-II [26]. Presence of depressive symptoms in people with chronic illnesses is defined as a score of 17 or more on the BDI-II [27].

In a short interview with a nurse, the patients answered three questions to screen for the presence of neurocognitive complaints (NCCs) [28]. Memory, planning and attention problems were surveyed. One or more “yes” answers are indicative for NCCs.

Clinical data were obtained through the electronic patient file.

### Analyses

Data were analyzed using the statistical software package SPSS, version 21 (SPSS, Inc., Chicago, IL). Categorical variables are reported as numbers and percentages, continuous variables as medians and interquartile ranges (IQR) since they were non-normally distributed. PHS and MHS were separately analyzed as continuous, dependent variables. For bivariate analysis, Mann–Whitney *U* tests and Kruskal–Wallis tests were used and correlations were calculated. Multivariable linear regression analysis was conducted to potentially identify determinants independently associated with HRQoL. Every variable with a *p*≤0.20 after bivariate analysis was put into a multivariable linear regression model. Backward conditional procedures were performed manually to identify independent determinants. These were sequentially put into a model, based on the enter procedure. Significance of the covariates is reported by the *p*-values, parameter estimates by the unstandardized coefficients (B) and 95% confidence intervals (CI). Goodness of fit was reported by the *R*^2^ of the model. Statistical significance was set at *p*≤0.05.

## Results

### Population

A total of 237 PLHIV participated in the study. Male–female ratio was 4:1, mean age was 45.8±10.7 years and 63.7% (144/226) of the participants were homosexual. Almost 90% (209/237) were Caucasian and 69.2% (164/237) were employed.

At the time of assessment, the vast majority (92.0%, 218/237) was receiving HAART, and viral load and CD4 cell count were < 40 copies/ml and >500 cells/µl in 74.3% (176/237) and 59.9% (142/237) of participants, respectively.

Depressive symptoms were observed in 22.3% (51/229) of the participants and 86 participants (36.3%) had NCCs.

In this sample, there is an underrepresentation of black PLHIV as compared to the PLHIV seen in the centre (10% vs. 24%) and of people with foreign origin as compared to the total Belgian cohort (10% vs. 49%). Male-to-female ratio is higher than the ratio in the total Belgian cohort (4 vs. 1.7). Mean age in the study sample (45.8 years) is higher than mean age of PLHIV seen in the centre (44.1 years) and mean age of the total Belgian cohort (43.1 years).

Population characteristics, also disaggregated by gender, are available as Supplementary file.

### Quality of life

Median PHS and MHS are 55.6 (IQR 48.2–60.6) and 52.0 (IQR 44.2–57.9), respectively. PHS is significantly higher than MHS (*p*<0.001, Table 1) and there is a significant correlation between them (0.644; *p*<0.01). The median scores on the subscales, for men and women, are shown in Table 1.

**Table 1:** Results of MOS-HIV: quality of life scores for men and women

	Median (IQR)	
	
	Men	Women
*Total scores*		
Physical health score	57.7 (49.6–60.9)	51.4 (44.2–54.5)
Mental health score	52.8 (45.1–58.7)	50.4 (41.6–54.9)
*Subscales*		
General health perceptions	70 (50–85)	63.9 (45–75)
Physical functioning	91.7 (75–100)	75 (50–83.33)
Role functioning	100 (100–100)	100 (50–100)
Social functioning	100 (80–100)	80 (60–100)
Cognitive functioning	80 (65–100)	75 (65–100)
Pain	88.9 (66.67–100)	77.8 (55.56–100)
Mental health	72 (56–80)	65.20 (56–76)
Energy	70 (55–80)	60 (45–75)
Health distress	85 (65–100)	70 (50–90)
Quality of life	75 (50–75)	75 (50–75)
Health transition	50 (50–75)	67.1 (50–75)

Table 2 shows the results of the bivariate analysis. A lower PHS and MHS is seen in PLHIV who are female (*p*<0.001 and 0.006), not working (seeking work <0.001 and 0.048, househusband/housewife 0.021 and 0.015, invalid both <0.001), living alone (*p*=0.003 and <0.001) and having an income <€1.500 (both *p*<0.001). Lower PHS and MHS are also associated with shorter time since diagnosis (*p*=0.017 and 0.040), the presence of depressive symptoms (both *p*<0.001), NCCs (both *p*<0.001), less satisfaction with relationship with physician (*p*=0.049 and 0.004), dissatisfaction with social support (both *p*<0.001), dissatisfaction with sex life (both *p*<0.001) and poor adherence (both *p*<0.001). Black ethnicity (*p*=0.030) and dissatisfaction with information received about medication (*p*=0.010) are associated with lower PHS, whereas not being married (divorced *p*=0.006, single <0.001) and drug use (*p*=0.021) are associated with lower MHS (Table 2).

**Table 2:** Results of bivariate analyses (Mann-Whitney *U* test, Kruskal–Wallis test or Pearson correlation)

Variable	PHS (median and IQR)	*p*	MHS (median and IQR)	*p*
Sex		<0.001		0.006
Female	51.37 (44.19–54.52)		50.37 (41.64–54.95)	
Male	57.65 (49.60–60.95)		52.83 (45.09–58.74)	
Sexual orientation (*n*=226)		0.066		0.155
Heterosexual	53.29 (44.56–59.60)		50.80 (43.09–56.40)	
Homosexual	57.65 (49.65–61.21)		53.02 (45.29–58.71)	
Bisexual	54.40 (44.64–59.13)		44.94 (41.10–53.54)	
Age (*correlation*)	−0.115	0.076	−0.092	0.158
Ethnicity (*n*=236)		0.030		0.680
White	57.16 (49.26–60.87)		52.57 (44.15–58.09)	
Black	52.27 (44.39–57.41)		50.52 (45.89–55.90)	
Viral load		0.651		0.238
< 50 copies/ml	56.53 (48.22–60.92)		51.92 (45.12–57.99)	
40–400 copies/ml	53.75 (49.55–58.93)		51.53 (43.90–56.47)	
401–1000 copies/ml	54.98 (45.74–59.08)		59.49 (54.69–62.54)	
>1000 copies/ml	55.65 (45.60–60.91)		51.87 (41.10–55.90)	
CD4 cell count		0.112		0.602
<200	45.87 (38.42–53.06)		52.93 (47.80–59.87)	
200–500	55.66 (47.53–60.23)		50.94 (44.15–56.16)	
>500	56.42 (49.70–60.87)		53.33 (44.24–58.19)	
ART treatment		0.464		0.602
No	54.01 (47.74–58.47)		52.42 (44.25–57.92)	
Yes	56.10 (48.23–60.73)		50.99 (39.50–58.13)	
Time since diagnosis (*correlation*)	−0.155	0.017	−0.133	0.040
Depressive symptoms		<0.001		<0.001
No	57.69 (51.99–60.95)		54.69 (50.26–58.89)	
Yes	47.82 (39.24–53.37)		39.76 (34.34–44.00)	
Civil status		0.095		0.003
Married	58.68 (53.71–61.41)		55.56 (49.95–60.13)	
Cohabitant	57.17 (48.21–60.87)		52.88 (43.38–60.71)	
Divorced	53.06 (45.99–59.08)		50.52 (45.12–54.82)	
Widow/widower	56.54 (51.26–60.22)		44.45 (37.84–52.99)	
Alone	53.74 (47.53–59.96)		50.52 (42.29–56.13)	
Domestic situation		0.003		<0.001
Living with partner/children, family or friends	57.71 (50.52–60.90)		53.47 (46.96–59.85)	
Living alone	53.24 (47.08–59.66)		50.37 (41.96–55.75)	
Place of residence		0.414		0.674
At home	56.21 (48.21–60.75)		52.20 (44.07–57.95)	
Others	53.06 (48.90–57.74)		50.52 (50.25–55.50)	
Children		0.086		0.184
No	57.24 (49.29–60.91)		52.75 (44.19–58.40)	
Yes	53.06 (44.58–59.45)		50.52 (45.04–55.52)	
Education		0.525		0.161
Primary school (12 years)	53.06 (44.19–58.60)		50.52 (41.78–59.19)	
College (15 years)	53.06 (46.81–59.47)		50.52 (43.90–55.03)	
College (18 years)	57.23 (48.23–60.87)		52.80 (45.54–60.13)	
College of higher education (3 years)	55.47 (50.21–60.73)		51.84 (45.89–55.90)	
College of higher education (>3 years)	58.87 (49.93–62.06)		51.95 (39.99–57.93)	
University	57.04 (52.96–59.08)		55.81 (46.81–58.34)	
Activity		<0.001		<0.001
Student	52.25 (44.19–55.52)		49.77 (45.89–54.06)	
Working	58.37 (53.06–61.24)		53.90 (48.98–58.65)	
Job seeker	49.30 (42.81–55.58)		47.05 (39.99–57.73)	
Housewife/househusband	43.05 (34.76–57.99)		45.24 (34.44–50.49)	
Invalid	40.90 (34.81–49.88)		41.43 (35.96–45.69)	
Retired	57.70 (51.65–58.66)		57.58 (47.80–60.71)	
Income (after taxes) (*n*=226)		<0.001		<0.001
<€ 1500	52.41 (41.67–58.74)		48.66 (40.66–55.51)	
≥€ 1500	57.95 (53.06–61.29)		54.26 (48.79–58.87)	
Smoking		0.816		0.200
No	58.89 (49.30–59.91)		52.61 (45.21–58.29)	
Yes	54.57 (46.28–61.05)		51.11 (42.95–56.73)	
Drug use		0.072		0.021
No	56.89 (49.26–60.75)		52.78 (45.20–58.09)	
Yes	50.66 (45.44–59.13)		48.41 (38.83–53.63)	
Alcohol use		0.075		0.112
No	54.44 (47.53–60.29)		50.52 (43.93–57.07)	
≤3/day	58.19 (49.88–61.29)		55.22 (45.75–59.19)	
>3/day	57.72 (45.12–59.07)		52.22 (37.16–57.73)	
Religion		0.350		0.857
Atheist	57.58 (50.93–60.75)		52.83 (44.19–58.19)	
Catholic	57.04 (48.23–60.87)		51.79 (44.25–58.19)	
Protestant	50.80 (48.10–54.52)		52.80 (46.01–55.50)	
Muslim	53.06 (51.09–63.49)		55.90 (50.52–56.40)	
Others	53.70 (45.41–59.96)		48.66 (43.90–57.60)	
Membership self-help group		0.585		0.248
No	55.66 (48.23–60.54)		52.57 (44.24–58.02)	
Yes	53.06 (45.99–59.83)		49.31 (44.98–51.25)	
Satisfaction relationship physician		0.049		0.004
Very satisfied	56.81 (49.31–60.80)		53.02 (45.16–58.62)	
Satisfied or indifferent	50.93 (45.41–58.96)		46.64 (39.88–53.70)	
Satisfied information medication (*n*=221)		0.010		0.558
No	49.55 (41.30–57.98)		50.25 (40.42–59.65)	
Yes	57.10 (49.33–60.87)		52.49 (45.09–57.92)	
Satisfaction support		<0.001		<0.001
(Very) Satisfied	57.64 (50.25–60.93)		53.52 (46.64–58.69)	
(Very) unsatisfied or indifferent	49.65 (42.04–57.18)		44.88 (35.96–50.94)	
Satisfaction sex life		<0.001		<0.001
(Very) satisfied	58.39 (53.06–61.22)		55.47 (50.49–59.85)	
(Very) unsatisfied or indifferent	51.09 (44.58–57.69)		45.54 (38.58–52.81)	
Neurocognitive complaints		<0.001		<0.001
No	58.25 (51.65–61.32)		55.41 (49.83–59.87)	
Yes	50.72 (44.20–56.10)		45.72 (39.61–51.79)	
SMAQ adherent (*n*=218)		<0.001		<0.001
No	49.70 (44.19–53.32)		48.41 (39.74–52.72)	
Yes	57.74 (50.80–61.07)		53.47 (45.89–58.87)	

Median and IQR are shown for categorical variables, the correlation coefficient for continuous variables.

Multivariable linear regression analysis shows that the presence of depressive symptoms, NCCs, work incapacity, satisfaction with the patient–physician relationship and non-adherence are independently associated with both poor PHS and MHS. Seeking work and being a househusband/housewife negatively influence PHS. Dissatisfaction with sex life is associated with lower MHS (Table 3).

**Table 3:** Multivariable linear regression models for PHS and MHS

	*p*	B	[95% Confidence Interval]
*PHS model*			
Constant	<0.001	56.04	[53.48; 58.60]
Depressive symptoms	0.017	−3.19	[−6.62; −1.75]
Work situation			
Student (vs. working)	0.158	−5.88	[−14.07; 2.31]
Seeking work (vs. working)	0.001	−6.54	[−10.50; −2.58]
Househusband/housewife (vs. working)	0.017	−8.74	[−15.90; −1.58]
Invalid (vs. working)	<0.001	−11.70	[−14.74; −8.67]
Retired (vs. working)	0.084	−3.47	[−7.0; 0.47]
Neurocognitive complaints	0.007	−2.88	[−4.981; −0.788]
Satisfaction with relation physician	0.028	2.85	[0.31; 5.40]
Non-adherence	0.001	−4.18	[−6.62; −1.75]
*R* ^2^=0.426			
*MHS model*			
Constant	<0.001	51.34	[49.01; 53.675]
Depressive symptoms	<0.001	−10.10	[−12.22; −7.98]
Work situation			
Student (vs. working)	0.153	−4.74	[−11.25; 1.77]
Seeking work (vs. working)	0.517	−1.04	[−4.20; 2.12]
Househusband/housewife (vs. working)	0.650	−1.32	[−7.03; 4.39]
Invalid (vs. working)	<0.001	−5.90	[−8.34; −3.45]
Retired (vs. working)	0.873	−0.26	[−3.39; 2.88]
Neurocognitive complaints	<0.001	−4.46	[−6.15; −2.77]
Satisfaction with sex life	<0.001	3.29	[1.64; 4.95]
Satisfaction with relation physician	<0.001	3.80	[1.77; 5.82]
Non-adherence	<0.001	−3.61	[−5.56; −1.67]
*R* ^2^=0.653			

MHS, mental health score; PHS, physical health score.

## Discussion

PHS and MHS of the MOS-HIV in this cohort of PLHIV are high and comparable to those found in HIV patients studied in other countries [10, 21, 29–31]. Scores on the subscales of the MOS-HIV are even comparable to those found in a general healthy population, except for a lower score for mental health [32]. The good clinical status of our cohort and the high representation of white gay men might be a potential explanation. In people with other chronic diseases such as chronic fatigue syndrome or rheumatoid arthritis, substantially lower HRQoL scores have been reported [32, 33]. It seems that HIV does not interfere with daily life to the same extent as these other chronic diseases.

Determinants associated with a lower HRQoL in this study are the presence of depressive symptoms, NCCs, working situation, satisfaction with the relationship with the physician, adherence and satisfaction with sex life.

It is well known that depressive symptoms negatively impact perceived HRQoL [16, 23, 34, 35]. In this study, depressive symptoms were independently associated with both poor MHS and PHS. Moreover, depressive symptoms interfere with all domains as measured by the subscales of the MOS-HIV (except health transition), which emphasizes their considerable impact. Systematic and periodic screening for depression, as well as an appropriate treatment if necessary, is imperative to intervene in this prominent problem.

NCCs were found to be independently associated with lower PHS, MHS and lower subscale scores (except health transition). Neurocognitive impairment has already been shown to be a predictor of poor HRQoL [36] and to be significantly associated with lower scores on all HRQoL subscales [37]. This might be explained by difficulties in coping with daily activities such as stocking, managing their money, planning appointments and so on, which potentially lead to frustration and a loss of independence [36]. Medical professionals, family members and PLHIV themselves should be vigilant for these signs. Screening and prevention of HIV-associated neurocognitive disorder should become more familiar in HIV care to determine effective treatment strategies (exclude neurotoxicity if possible, treating co-morbidity, etc.) [38].

We could not, however, confirm the presence of NCCs by a neurocognitive testing battery, but it was previously observed that patient-reported measures of neurocognitive functioning and results on objective neurocognitive testing correlate significantly [37].

Several studies report that employed PLHIV tend to have better HRQoL [10, 12, 39–41]. Employment has an important role in the daily life of people. It provides structure, a social support network, role identity and meaning [40]. Physical functioning, role functioning, social functioning, pain, mental health and health distress were found to be significantly affected by disability. Differences in physical health as compared to employed people were also observed in job seekers. They have lower scores in physical-, role-, and social functioning and energy.

The exact association between employment and HRQoL remains unproven. This is possibly a bi-directional relationship [10, 12]. PLHIV with good HRQoL can choose to work (selection hypothesis), or work can be a source of wellbeing (causation hypothesis) [10]. Working should be encouraged and therefore possible internal or external barriers should be addressed [42]. PLHIV could be advised by peers, medical professionals and HR- and labour legislation experts about their rights and about practical concerns and discomforts. Our research group, in collaboration with the Flemish expertise centre on sexual health, currently gathers experiences from PLHIV at the workplace. This is a first step in developing a framework for employers and employees to inform them and to guide them through HIV at the workplace.

The importance of a good patient–physician relationship is emphasized by its contribution to both physical and mental health. It was shown that a good and trustful patient–physician relationship contributes to a good adherence [43, 44] and to an undetectable viral load [44]. Mental health improves by the feeling of “being treated as a person” and trust in the physician [44, 45]. The perception of empathy and a good knowledge about HIV seem to be predictive for satisfaction about the patient–physician relationship [46]. Physicians should be aware of this and invest in their relationship with PLHIV.

Furthermore, adherence itself influences HRQoL. Differences between adherent versus non-adherent participants are seen in all subscales of the MOS-HIV, which corresponds with the overall conclusion of the review on this topic by Geocze, Mucci, De Marco, Nogueira-Martins and Cicero [20]. Out of 12 studies, 10 showed a positive association between adherence and HRQoL. A causal relationship was, however, not clear. Adherence to ART could lead to better HRQoL, considering the protective role of ART in disease progression and mortality. However, ART frequently causes serious side effects (e.g. nausea, diarrhoea, lipodystrophy), which of course negatively influence HRQoL. Some PLHIV may therefore feel the tendency to stop adhering to ART in an attempt to re-establish their HRQoL. Another possibility could be that adherence and HRQoL have overlapping determinants. However, it is evident that adherence remains a highly important goal and moreover, interventions to improve adherence could have additional HRQoL benefits.

PLHIV who are satisfied about their sex life have a higher MHS than those who are not. In the literature, studies addressing this relationship are scarce. Most research discusses sexual dysfunction rather than sexual satisfaction [47]. However, there is evidence that a good sex life contributes to good HRQoL in the general population [48] and that experiencing sexual problems is associated with poorer quality of life in gay men [49]. In our study, dissatisfaction with sex life is associated with significant lower scores on all subscales of the MOS-HIV except health transition. Apparently, multiple domains of HRQoL are influenced by this presently understudied outcome measure. We recommend to break the taboo about sexual problems and to take these complaints seriously. They can be the expression of multiple stressors: for example, troubles in the current relationship, loneliness, trauma and physical causes. Accurate identification of the cause(s) should lead to the right treatment approach [50].

There are several study limitations that need to be addressed. The number of participants is limited. Nevertheless, good predictive models for HRQoL were achieved and the number is considered as adequate given the great detail in our research. A second limitation is the self-report of depressive symptoms and NCCs. Formal testing by a psychiatrist or neuropsychologist was not done. Because of the cross-sectional design, we were not able to determine causal relationships. Furthermore, taking into account the non-normal PHS and MHS, linear regression analysis reveals less correct models than median regression analysis would do. Our study results can also not be generalized to the population of PLHIV in Belgium, as our sample included an overrepresentation of gay men, Belgian and older PLHIV. Finally, the use of separate models for physical and mental health may be artificial, regarding the evidence about a strong, bi-directional relationship between them [51]. In fact, in this study, physical and mental health were found to be influenced by mainly the same parameters.

Behind these limitations, some strengths of this pilot study should be considered. To the best of our knowledge, this study is the first to investigate HRQoL among PLHIV in Belgium. Moreover, in addition to clinical variables, whose relationship with HRQoL has been the focus of a limited number of studies [6, 52, 53], many other (socio-economic, behavioural and psychological) variables were analyzed and were found to be associated with HRQoL. Calling more attention to non-clinical parameters in HIV care seems to be a relevant approach.

## Conclusions

PHS is high in this cohort of PLHIV. Not the clinical, but socio-economic (working), behavioural (adherence, sex life) and (neuro)psychological (depressive symptoms, NCCs) variables significantly influence HRQoL, as well as the perceived quality of the patient–physician relationship. Future research should focus on interventions to improve HRQoL, in hospital settings (supporting adherence, screening for and treating depression and neurocognitive problems, improving patient–physician relationship) and abroad (framework for HIV at the workplace, promoting sexual health).
